# Antihypertensive Effect of Radix Paeoniae Alba in Spontaneously Hypertensive Rats and Excessive Alcohol Intake and High Fat Diet Induced Hypertensive Rats

**DOI:** 10.1155/2015/731237

**Published:** 2015-02-16

**Authors:** Chen Su-Hong, Chen Qi, Li Bo, Gao Jian-Li, Su Jie, Lv Gui-Yuan

**Affiliations:** ^1^Zhejiang Chinese Medical University, Hangzhou, Zhejiang 310053, China; ^2^Wenzhou Medical University, Wenzhou, Zhejiang 325035, China; ^3^The Third People's Hospital of Hangzhou, Hangzhou 310009, China

## Abstract

Radix Paeoniae Alba (Baishao, RPA) has long been used in traditional Chinese medicine formulation to treat hypertension by repression the hyperfunction of liver. However, whether the RPA itself has the antihypertensive effect or not is seldom studied. This study was to evaluate the protective effect of RPA on hypertensive rats. Alcohol in conjunction with a high fat diet- (ACHFD-) induced hypertensive rats and spontaneously hypertensive rats (SHR) was constantly received either RPA extract (25 or 75 mg/kg) or captopril (15 mg/kg) all along the experiments. As a result, RPA extract (75 mg/kg) could significantly reduce systolic blood pressure of both ACHFD-induced hypertensive rats and SHR after 9-week or 4-week treatment. In ACHFD-induced hypertensive rats, the blood pressure was significantly increased and the lipid profiles in serum including triglyceride, total cholesterol, LDL-cholesterol, and HDL-cholesterol were significantly deteriorated. Also, hepatic damage was manifested by a significant increase in alanine transaminase (ALT) and aspartate transaminase (AST) in serum. The RPA extract significantly reversed these parameters, which revealed that it could alleviate the liver damage of rats. In SHR, our result suggested that the antihypertensive active of RPA extract may be related to its effect on regulating serum nitric oxide (NO) and endothelin (ET) levels.

## 1. Introduction

China is undergoing remarkable economic development, social reform, and globalization with rapidly changing lifestyles in recent years, which has resulted in alarming prevalence of hypertension. Hypertension and prehypertension were significantly associated with increased all-cause and cardiovascular mortality (*P* < 0.0001). In 2005, 2.33 million (95% CI 2.21–2.45) cardiovascular deaths were attributable to increased blood pressure in China [1]. Worldwide, the number of adults with hypertension in 2025 is predicted to increase by about 60% to a total of 1.56 billion [2]. The factors contributing to the development of hypertension are various, and dietary habit undoubtedly is an important one.

The association between alcohol consumption, blood pressure, and the prevalence of hypertension has been studied in the past 20 years. The relationship between light-to-moderate alcohol consumption (1-2 standard wine drinks a day) and the incident of hypertension remains controversial [3], but excessive alcohol intake dose elevates blood pressure, with global estimates that the attributable risk for hypertensive disease from alcohol is 16% [4]. In the UK, the accepted alcohol limits are 16 grams a day for women—the alcohol in just over a medium glass of wine—and 32 grams for men, roughly half a bottle of red wine.

Recent epidemiological studies further reveal that the high-fat diet is also strongly associated with the development and aggravation of hypertension and chronic target organ damage [5, 6]. In addition, it has been reported that combined consumption of a high fat diet with alcohol can aggravate obesity and liver fat accumulation [7, 8].

It is a tradition that herbal medicine, such as Radix Paeoniae Alba (RPA),* Chrysanthemum*, and* Uncaria rhynchophylla*, is used in the treatment of hypertension in China. Among them, RPA is the major constituent material of many complex preparations (mixture formulation of herbal drugs) to treat hypertension [9]. RPA, the dry root of* Paeonia lactiflora* Pall, is composed of nearly 10 types of medicinal substances, mainly paeoniflorin. According to the scientific research, RPA had wide variety of pharmacological actions such as anti-inflammatory, antioxidation, and strengthening immunity [10–12]. Qian et al. found that paeoniflorin, the main active ingredient in the root of Paeonia Radix, inhibited the proliferation of pulmonary artery smooth muscle cells (PASMCs) by blocking cell cycle progression in the S phase through activation of A2BAR under hypoxic conditions [13]. Ethanol extract of* Paeonia lactiflora* Pall relaxes vascular smooth muscle via endothelium-dependent and Akt- and SOCE-eNOS-cGMP-mediated pathways through activation of both K(Ca) and K(ATP) channels and inhibition of L-type Ca^2+^ channels [14].

To further clarify the antihypertensive mechanisms of RPA, the present study was designed to investigate the antihypertensive effect of RPA, both in the alcohol in conjunction with a high fat diet- (ACHFD-) induced hypertension model and spontaneously hypertensive rat (SHR) model. Furthermore, we also investigate the relationship between its protection effect in ACHFD induced liver damage and its antihypertensive effect.

## 2. Materials and Methods

### 2.1. Chemicals

Alcoholic drink (Alcohol = 52%, Red Star Corp, Beijing, China) was diluted with distilled water to alcohol = 23% (v/v). Biochemical reagents, such as total cholesterol (TC), triglyceride (TG), high density lipoprotein-cholesterol (HDL-C), low density lipoprotein-cholesterol (LDL-C), alanine transaminases (ALT), and aspartate transaminases (AST), were purchased from MeiKang Chemical Co. (Ningbo, Zhejiang, China). ELISA kits, such as plasma renin activity (PRA), angiotensin II (Ang II), aldosterone (ALD), and endothelin-1 (ET-1), were all purchased from HengYuan Chemical Co. (Shanghai, China). Nitric oxide (NO) kit was purchased from Nanjing Technology Co., Ltd. (JiangSu, China). The ethanol extract of RPA was obtained from Zelang Medical Technolody Co. (Nanjing, Jiangsu, China). The paeoniflorin in the extract was 53% detected by HPLC analysis.

### 2.2. Animal Groups

Male SD rats weighing 190–220 g were obtained from Animal Supply Center of Zhejiang Academy of Medical Science (Hangzhou, China). Male normotensive Wistar Kyoto (WKY) and spontaneously hypertensive rats (SHR) weighing 300–350 g were obtained from Vital River Laboratory Animal Inc. (Beijing, China). All the animals were housed in standard environmental conditions at a temperature of 25 ± 1°C, humidity of 55 ± 5%, and a 12/12 h light/dark cycle and allowed a standard pellet chow diet and water ad libitum for 1 week. All procedures were performed according to protocols following the guidelines for the Use and Care of Laboratory Animals published by the Zhejiang province (2009).

### 2.3. Animal Treatments

SD rats were firstly divided into 5 groups (*n* = 7). The normal group received a standard pellet chow diet and water throughout the first 8 weeks of the experiment. The ACHFD-induced hypertensive rats, given high fat diet and 23% alcohol-water drinking for 8 weeks, have a ≥15% increase in systolic blood pressure (SBP). Daily water consumption was estimated individually for every animal 1 week before the experiment. During the experiment, water consumption was controlled. This protocol was adopted based on our preliminary studies [15]. For the next 8 weeks, group 2 rats were set as the model control group and continued to receive a high fat diet and alcohol; group 3 rats were given a high fat diet, alcohol, and captopril; groups 4 and 5 rats were given a high fat diet, alcohol, and RPA extract (at the doses of 25 and 75 mg/kg, p.o.). SHRs were randomly assigned to four groups of eight rats each. The first group was set as SHR control group; the second group received captopril; Groups 3 and 4 received RPA extract (at the doses of 25 and 75 mg/kg, p.o.). Throughout the experiment, body weight was evaluated.

### 2.4. Blood Pressure Measurement

Blood pressure, which included SBP, diastolic blood pressure (DBP), and mean arterial blood pressure (MBP), was measured by a noninvasive method of tail-cuff plethysmography (Shanghai Alcott Biotech Co., Ltd., Shanghai China) every week. To get accurate blood pressure, at least 6 consecutive determinations were recorded.

### 2.5. Biochemical Analysis

At the end of experiment, the ACHFD-induced hypertensive rats were fasted overnight and anesthetized with pentobarbital. Blood was drawn from the ophthalmic venous plexus and centrifuged at 3000 rpm for 10 min. The concentrations of TC [16], TG, LDL-C, and HDL-C were determined by the ACCUTE (TBA-40FR) automatic biochemical analyzer (Toshiba, Japan). The activities of ALT and AST were also determined by TBA-40FR using the method of IFCC [17].

### 2.6. Determination of PRA, Ang II, ALD, and ET-1 Activities

At the end of experiment, the SHR were fasted overnight and anesthetized with pentobarbital. Blood was drawn from the ophthalmic venous plexus and centrifuged at 3000 rpm for 10 min. Serum was separated to determine plasma renin activity (PRA), angiotensin II (Ang II), aldosterone (ALD), and endothelin-1 (ET-1) activities by the method of enzyme linked immunosorbent assay [18]. All of the procedures were performed as described in the assay kit. Briefly, the various samples for ELISA measurement were diluted in 0.1% BSA/Tris-buffered saline and were incubated in a 96-well plate precoated with a capture antibody directed against PRA, Ang II, ALD, or ET-1 for 30 min at 37°C. The wells were then washed five times in 0.05% Tween 20/PBS and incubated with a secondary antibody against PRA, Ang II, ALD, or ET-1 conjugated to horseradish peroxidase for an additional 30 min at 37°C. The plates were then washed again five times, substrate solution containing H_2_O_2_ and tetramethylbenzidine (TMB) was added, and optical density was determined at 450 nm. All assays were done in duplicate, and the protein levels were calculated using a standard curve derived from known concentrations of the respective recombinant proteins.

### 2.7. Determination of Serum NO Level

NO has a brief half-life and is rapidly converted to the stable end-products NO_2_^−^ and nitrate (NO_3_^−^) in typical oxygenated aqueous solutions [19]. Therefore, the serum NO level was evaluated by nitrate reductase method [20]. Blood sample was centrifuged at 3000 rpm for 10 min and serum was separated to determine NO concentration. The reductase nitrate restores all NO_3_^−^ to NO_2_^−^, which can be determinate by the excellent colorimetric reagent to measure total nitrite as an indicator of NO production as described before. All of the procedures were performed as described in the assay kit.

### 2.8. Statistical Analysis

All measurements were expressed as the mean ± standard deviation and subjected to one-way analysis of variance (ANOVA), followed by Fisher's least significant difference (LSD) comparison. *P* value of <0.05 was considered statistically significant. All analyses were performed using an updated version of SPSS software.

## 3. Results

### 3.1. Effects of Radix Paeoniae Alba Extract on Body Weight

The effects of the RPA extract on the weight of ACHFD-induced hypertensive rats (*n* = 7) or SHR (*n* = 8) were compared with those of rats fed a control diet (Table 1). We found that PRA did not affect the body weight of the rats in both hypertensive models. Although it has been reported that excessive alcohol consumption combined with high fat diet results in weight gain and metabolic disorders [21, 22], the different result from our experiment may be explained by anorexia phenomenon of the high fat rats and the empty calories caused by alcohol consumption [7].

### 3.2. Effects of Radix Paeoniae Alba Extract Supplement on Blood Pressure of ACHFD-Induced Hypertensive Rats

**Figure 1:**
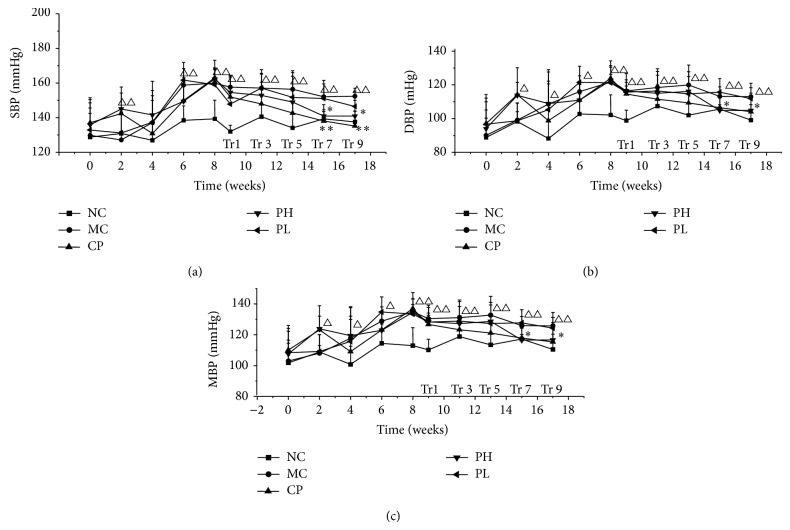
SBP, DBP, and MBP changed in ACHFD-induced hypertensive rat model establishment period (0–8 week) or treatment period (9–17 week). Values are expressed as the mean ± SD (*n* = 7). (^△^ *P* < 0.05, ^△△^ *P* < 0.01 versus normal control, ^*^ *P* < 0.05, ^**^ *P* < 0.01 versus model group.) SBP: systolic blood pressure; DBP: diastolic blood pressure; MBP: mean blood pressure; NC: normal control group; CP: captopril group; MC: model control group; PH: RPA extract high dose group (75 mg/kg); PL: RPA extract low dose group (25 mg/kg); Tr: treatment.

Before the experiment, there were no significant differences in SBP, DBP, and MBP among the groups. During the period of model establishment, SBP, DBP, and MBP of the normal group (NC) were constant while the administration of alcohol and high fat diet significantly increased the SBP, DBP, and MBP of all the model groups (Figure 1(a)). The RPA extract groups presented a downtrend line, which is meaningful. After 9 weeks of treatment with RPA, the SBP of the RPA high dose group (PH) was reduced from 162 mmHg to 141 mmHg and was significant lower than that of the model control group (MC). On the other hand, only captopril could significant reduce SBP, DBP, and MBP of ACHFD-induced hypertensive rats after 7-week treatment (Figures 1(b) and 1(c)).

### 3.3. Effects of Radix Paeoniae Alba Extract Supplement on Lipid Profile

As shown in Table 2 the concentrations of TC and LDL-cholesterol were significantly higher while the concentration of HDL-cholesterol was significantly lower in the rats fed with high fat diet and alcohol, compared with the normal diet group. This morbid state was significantly prevented by the treatment with the plant extract at the dose of 75 mg/kg. At the same dose, the increase of atherogenic index was also prevented as compared to untreated rats. The circulating concentration of TG was not changed significantly by the high fat diet and alcohol consumption.

### 3.4. Effects of Radix Paeoniae Alba Extract Supplement on Liver Function

The effects of RPA extract on liver function in ACHFD-induced hypertensive rats (*n* = 7) or SHR (*n* = 8) are summarized in Table 2. Chronic alcohol and high fat administration resulted in a greatly increased AST and ALT concentration in serum, as compared to normal rats. Upon RPA (low dose) treatment, levels of ALT and AST significantly decreased as compared to untreated hypertensive rats.

### 3.5. Effects of Radix Paeoniae Alba Extract Supplement on Blood Pressure of the SHR

**Figure 2:**
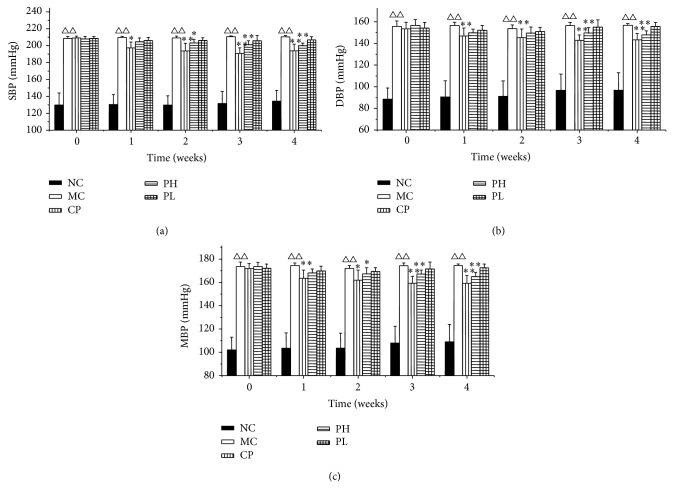
SBP, DBP, and MBP during 4 weeks of treatment in the SHR. Values are expressed as the mean ± SD (*n* = 8). (^△^ *P* < 0.05, ^△△^ *P* < 0.01 versus normal control, ^*^ *P* < 0.05, ^**^ *P* < 0.01 versus model group.) SBP: systolic blood pressure; DBP: diastolic blood pressure; MBP: mean blood pressure; NC: normal group; CP: captopril group; MC: model group; PH: RPA extract high dose group (75 mg/kg); PL: RPA extract low dose group (25 mg/kg).

Changes in SBP, DBP, and MBP of the SHR during 4 weeks of administration are shown in Figure 2. Before treatment, SBP, DBP, and MBP were significantly (*P* < 0.01) higher in the SHR control than in the WKY control. Since the third week of treatment, there was a significant suppression of SBP, DBP, and MBP (*P* < 0.01) in PH-treated SHR compared to the SHR control. In the fourth week, the SBP of the RPA high dose group (PH) was reduced from 208 mmHg to 200 mmHg and was significantly lower than that of the model control group (MC). The positive control drug, captopril, also significantly lowered SBP, DBP, and MBP in the SHR.

### 3.6. Effects of Radix Paeoniae Alba Extract Supplement on Renin-Angiotensin-Aldosterone System (RAAS) Activities

**Figure 3:**
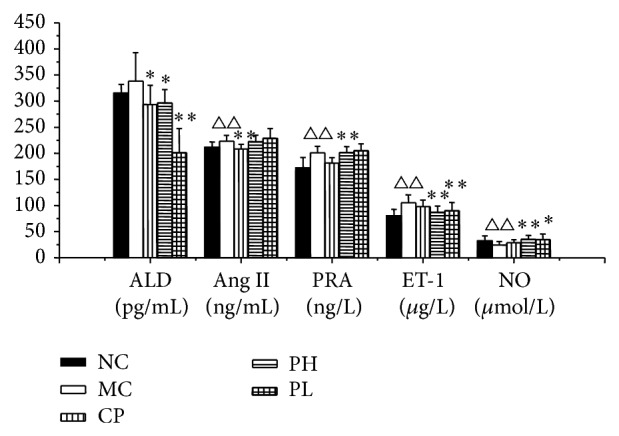
Serum PRA, Ang II, ALD, NO, and ET-1 concentration after four weeks of treatment. Values are expressed as the mean ± SD (*n* = 8). (^△^ *P* < 0.05, ^△△^ *P* < 0.01 versus normal control, ^*^ *P* < 0.05, ^**^ *P* < 0.01 versus model group.) NC: normal control group; CP: captopril group; MC: model control group; PH: Radix Paeoniae Alba extract high dose group (75 mg/kg); PL: Radix Paeoniae Alba extract low dose group (25 mg/kg).

The results of plasma renin activity (PRA), angiotensin II (Ang II), and aldosterone (ALD) activity analysis after feeding for 4 weeks are shown in Figure 3. As a result, RPA extract treatment caused a significant decrease in serum ALD levels but presented no significant influence in the Ang II and PRA activity in the serum.

### 3.7. Effects of Radix Paeoniae Alba Extract Supplement on NO and ET-1 Concentration

The serum nitric oxide (NO) and endothelin-1 (ET-1) measurements are presented in Figure 3. The serum NO concentration in the treatment groups was significantly higher than that in the SHR control group. This indicates that the oral administration of RPA extract increased the serum NO concentration. Also, the addition of this herbal extract markedly suppressed the increased concentration of serum ET-1 concentration.

## 4. Discussion

In traditional Chinese medicine, the overactive liver function is 240 recognized as a major cause of hypertension, and Radix Paeoniae Alba could have an antihypertensive effect by repressing the hyperfunction of liver. In this study, we observed antihypertensive properties of the RPA extract in two different models of hypertension (spontaneous or alcohol and high fat diet-induced), its mechanism may be related to the lipid lowering, liver protection, and endothelial function improvement.

Obesity, diet, and alcohol consumption constitute major environmental determinations of blood pressure elevation [23]. In the present study, alcohol and high fat feeding significantly increased total cholesterol, LDL-cholesterol, and the atherogenic index and decreased HDL-cholesterol. It followed by the blood vessel damage caused by fat deposits [24]. Chronic alcohol drinking with high-fat consumption led to the abnormal lipid metabolism and liver damage, as well as increase of SBP in rats. Therefore, we strongly propose that long term consumption of alcohol with a high fat diet could produce an appropriate model for recent excessive nutritional and alcohol abuse.

In this study, RPA extract (75 mg/kg) significantly prevented the increase of total cholesterol, LDL-cholesterol, atherogenic index, and the decrease of HDL-cholesterol and also significantly prevented the abnormal drop of HDL-cholesterol/TC ratio. HDL-cholesterol/TC ratio is a superior measure of cardiovascular diseases risk than non-HDL-cholesterol. It captures the protective effect of HDL-cholesterol as well as the harmful effects of non-HDL-cholesterol in a single parameter and remains one of the best dyslipidaemic measures [25]. Moreover, significant increases in the serum levels of ALT and AST are conventional indicators of liver damage [26]. Administration of the RPA extract tended to attenuate the increased concentrations of serum ALT and AST.

Several experiments have linked alcohol ingestion, high fat diet, and hypertension via a mechanism of elevated tissue angiotensin II levels, depletion of the endothelial nitric oxide generating system, and overproduction of endothelin-1 [27, 28]. NO dilates blood vessel and inhibits platelet aggregation and proliferation of vascular smooth muscle cells [29]. The decrease of NO concentration in the blood may help explain the vascular pathology of hypertension. Based on the fact that angiotensin II increases eNOS expression and endothelial NO production by activating the angiotensin II type 2 receptor [30], Kleinhenz et al. suggested that ethanol-induced increases in the activity of the renin-angiotensin system contribute to enhanced vascular eNOS expression and NO production [31]. The link between NO availability and the metabolic adaptation to a high fat diet was also studied by Razny et al. [28]. They suggested that increased NO availability attenuates some high fat diet induced alterations in metabolism. In our study of spontaneously hypertensive rats, the serum concentration of NO in the normotensive controls was significantly higher than that in the hypertensive controls. The administration of RPA extract significantly increased serum NO concentration. However, there was no difference in serum Ang II concentration among the groups.

In contrast to NO, ET-1 is a powerful vasoconstrictor that has been demonstrated to mediate increased blood pressure. ET-1 exerts its effects through the activation of ET_A_ and ET_B_ receptors [32, 33]. ET_A_ receptors are present on vascular smooth muscle cells, whereas ET_B_ receptors are located predominantly on endothelial cells. ET_A_ receptor activation results in vasoconstriction and smooth muscle proliferation, whereas ET_B_ receptor binding stimulates nitric oxide (NO) synthesis, resulting in vasodilation [34]. In a model of visceral obesity and hypertension, da Silva et al. find that a long-term high fat diet may cause visceral obesity and increased arterial pressure and ET-1 appears to play an important role in the maintenance of arterial pressure [35]. In our study, RPA extract significantly inhibited the abnormal increase of the serum ET-1 level in spontaneous hypertension rats. Combined with previous results, we suggest that RPA extract might decrease the higher endothelin-1 levels and stimulated the ET_B_ receptors which finally resulted in the massive release of NO.

The ancient literature Kai Bao Ben Cao has mentioned the potential vasoprotective properties of Radix Paeoniae Alba (Baishao). Here, our present study proves that the antihypertension effect of RPA may result from its liver protection activity and its improvement of endothelial function by reducing ET-1 and increasing NO concentrations.

## Figures and Tables

**Table 1:** Effect of Radix Paeoniae Alba extract on the body weight of rats.

Group	Parameter	Initial weight (g)	Final weight (g)	Weight gain (g)
SD normal (*n* = 7)		245.50 ± 15.69	423.00 ± 26.05	178.38 ± 26.05
ACHFD rats (*n* = 7)	control	246.10 ± 19.47	427.00 ± 12.18	181.75 ± 19.59
	cap	237.40 ± 8.06	426.00 ± 23.71	189.43 ± 21.02
	PH	243.90 ± 13.98	420.67 ± 38.50	175.78 ± 38.32
	PL	244.30 ± 9.57	417.00 ± 28.52	170.78 ± 33.40

WKY normal (*n* = 8)		328.00 ± 24.54	343.13 ± 23.50	15.13 ± 28.80
SHR control (*n* = 8)		335.88 ± 23.13	333.25 ± 16.20	−2.63 ± 20.58
SHR cap (*n* = 8)		329.25 ± 24.50	349.25 ± 32.88	20.00 ± 14.73
SHR PH (*n* = 8)		332.38 ± 24.85	342.50 ± 27.32	10.13 ± 30.65
SHR PL (*n* = 8)		342.63 ± 27.24	353.50 ± 29.17	10.88 ± 20.41

ACHFD: Alcohol in conjunction with a high fat diet; cap: captopril; PH: RPA extract high dose group (75 mg/kg); PL: RPA extract low dose group (25 mg/kg).

**Table 2:** Effect of Radix Paeoniae Alba extract on the serum lipid profile and liver functions parameters.

Group	Parameter	TC (mmol/L)	TG (mmol/L)	HDL-C (mmol/L)	LDL-C (mmol/L)	HDL-C/TC ratio	AI	AST (U/L)	ALT (U/L)
SD normal (*n* = 7)		2.11 ± 0.31	0.53 ± 0.12	0.71 ± 0.09	0.87 ± 0.07	0.34 ± 0.02	1.98 ± 0.18	256.29 ± 42.94	77.43 ± 11.10
ACHFD rats (*n* = 7)	control	3.83 ± 0.49^ΔΔ^	0.70 ± 0.23	0.46 ± 0.08^ΔΔ^	1.71 ± 0.29^ΔΔ^	0.12 ± 0.03^ΔΔ^	7.79 ± 2.71^ΔΔ^	323.14 ± 113.79	161.43 ± 90.83^Δ^
	cap	3.68 ± 0.65	0.45 ± 0.15^*^	0.39 ± 0.07	1.71 ± 0.32	0.11 ± 0.03	8.61 ± 2.40	302.71 ± 115.17	180.00 ± 108.25
	PH	2.87 ± 0.52^**^	0.80 ± 0.38	0.60 ± 0.13^*^	1.18 ± 0.19^**^	0.21 ± 0.04^**^	3.89 ± 0.80^**^	322.57 ± 86.41	145.57 ± 58.26
	PL	3.86 ± 0.54	0.72 ± 0.16	0.45 ± 0.09	1.75 ± 0.28	0.12 ± 0.03	7.96 ± 2.50	211.43 ± 58.66^*^	84.00 ± 32.19^*^

WKY normal (*n* = 8)		1.81 ± 0.19	0.65 ± 0.23	0.68 ± 0.09	0.36 ± 0.11	0.37 ± 0.04	1.69 ± 0.30	250.88 ± 37.69	69.50 ± 15.52
SHR control (*n* = 8)		2.23 ± 0.44^Δ^	0.87 ± 0.22^Δ^	0.59 ± 0.06^Δ^	1.33 ± 0.52^ΔΔ^	0.28 ± 0.07^ΔΔ^	2.83 ± 0.95^ΔΔ^	297.50 ± 39.42^Δ^	109.63 ± 39.94^Δ^
SHR cap (*n* = 8)		2.57 ± 0.63	0.76 ± 0.12	0.62 ± 0.13	1.49 ± 0.48	0.24 ± 0.04	3.21 ± 0.74	282.33 ± 80.52	108.14 ± 31.24
SHR PH (*n* = 8)		2.44 ± 0.46	0.61 ± 0.09	0.69 ± 0.10^*^	1.28 ± 0.21	0.28 ± 0.02	2.54 ± 0.28	285.75 ± 48.38	91.13 ± 13.47
SHR PL (*n* = 8)		2.72 ± 0.64	0.75 ± 0.23	0.66 ± 0.10	1.48 ± 0.26	0.25 ± 0.03	3.09 ± 0.54	322.63 ± 90.36	88.25 ± 17.55

TC: total cholesterol; TG: triglyceride; HDL-C: high-density lipoprotein cholesterol; LDL-C: low-density lipoprotein cholesterol; atherogenic index = (TC-HDL-C)/HDL-C (^Δ^ *P* < 0.05, ^ΔΔ^ *P* < 0.01 versus normal control, ^*^ *P* < 0.05, ^**^ *P* < 0.01 versus model group). ACHFD: Alcohol in conjunction with a high fat diet; cap: captopril; PH: RPA extract high dose group (75 mg/kg); PL: RPA extract low dose group (25 mg/kg).
